# Robotic automation and unsupervised cluster assisted modeling for solving the forward and reverse design problem of paper airplanes

**DOI:** 10.1038/s41598-023-31395-0

**Published:** 2023-03-14

**Authors:** Nana Obayashi, Kai Junge, Stefan Ilić, Josie Hughes

**Affiliations:** CREATE Lab - Computational Robot Design & Fabrication Lab, EPFL, 1015 Lausanne, Switzerland

**Keywords:** Mechanical engineering, Computer science

## Abstract

Although often regarded a childhood toy, the design of paper airplanes is subtly complex. The design space and mapping from geometry to distance flown is highly nonlinear and probabilistic where a single airplane design exhibits a multitude of trajectory forms and flight distances. This makes optimization and understanding of their behavior challenging for humans. By understanding the behavior of paper airplanes and predicting flight behavior, there is a potential to improve the design of aerial vehicles that operate at low Reynolds numbers. By developing a robotic system that can fabricate, test, analyze, and model the flight behavior in an unsupervised fashion, a wide design space can be reliably characterized. We find there are discrete behavioral groups that result in different trajectories: nose dive, glide, and recovery glide. Informed by this characterization we propose a method of using Gaussian mixture models to extract the clusters of the design space that map to these different behaviors. This allows us to solve both the forward and reverse design problem for paper airplanes, and also to perform efficient optimization of the geometry for a given target flight distance.

## Introduction

The design or structure of many biological systems exploit natural phenomena to manifest spontaneous behaviors of considerable complexity. This includes autorotating or burrowing seeds^1,2^, vortex-based propulsion^3^, and laminar boundary layer assisted drag reduction^4^. These all leverage interactions with fluids to achieve diversity in behavior, efficiency or other benefits. Driven by the inherent nonlinear dynamics^5–7^, the behavior of such systems can often appear random in their evolution and show multiple possible states or behaviors. Evolution has enabled the design of these systems which show complex emergent behaviors over significant periods of time^8^, and this optimization continues till this day. Developing means of artificially designing systems which exploit these nonlinear dynamics could allow for similarly complex behavior to be realized^9^, enabling improvements in the performance of system behaviors, be it locomotion^10^, flying, or other resultant or emergent behaviors^11^.

One such design system that seeks to exploit nonlinear physical phenomena is that of paper airplanes^12^. Outwardly a simple ‘toy’^13^, they show complex aerodynamic behaviors which are most often overlooked. This is a design space where flat deformable paper is constructed into a structure which often mimics the shape of a fixed-wing aircraft or a dart enabling gliding behavior. When launched, there are resulting complex physical interactions between the deformable paper structure and the surrounding fluid leading to a particular flight behavior. The resulting flight trajectories show discrete behavioral groups which are characterized by the shape of their trajectory; some nose dive, others glide, and others show a recovery glide. These discrete behavioral groups are widely seen across gliding, falling or flying paper structures^14–17^. A single airplane design can also show multiple behaviors on different launches, resulting in a probabilistic behavior and also considerable variation in resultant flight trajectory and corresponding flight distance. Resulting from the quest for efficient micro air vehicles (MAVs)^18,19^, there is a focus on understanding the behaviors of paper airplanes as they fly in the same Reynolds number range, but have a much simpler form than MAVs. A number of data-driven and analytical approaches have been explored to understand or predict the resulting flight path or behavior. Experimental analysis in flow channels has enabled flow visualizations and force measurements for specific dart morphology airplanes^20^. Later studies focused on the change in lift coefficient with the sweep angle of dart planes at low Reynolds numbers^21^. The effect of changing morphology or structure has also been addressed by investigating the transformation of falling paper into a paper glider by changing the location of the center of mass^22^ and also by varying the span-wise flexibility of tumbling wings^23^. Simulation driven CFD analysis is an alternative approach which has enabled the exploration of a wider design space. For three different airplane geometries the resultant lift and drag coefficients^12^ were determined as a means of optimizing aerodynamic efficiency. Computational analysis has also provided a tool for simulating the flight dynamics for a sample of designs, yet this tool has remained fully theoretical and does not capture the observed stochasticity^24^. Despite the insights gained from these experimental and simulation studies the range of designs explored is limited. Furthermore, the identification of a comprehensive and universal analytical solution to predict the resultant flight behavior or aerodynamic performance from the design of the airplanes has not yet been demonstrated.

**Figure 1:**
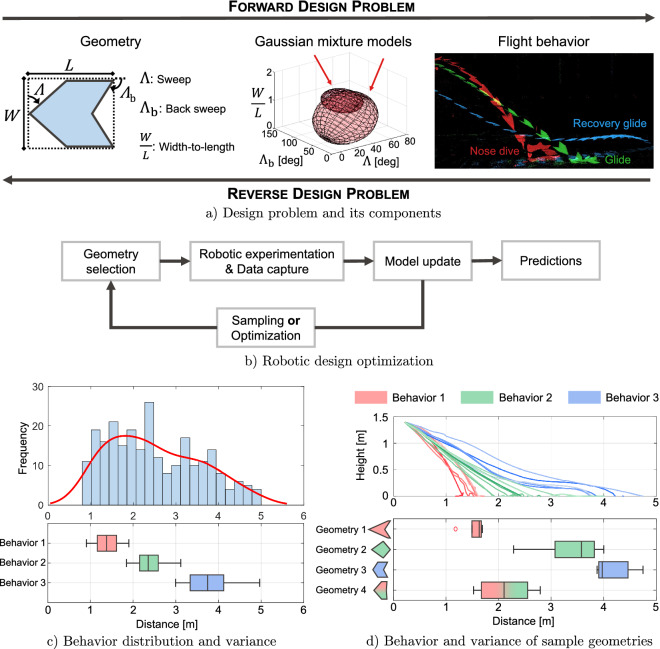
(**a**) Summary of our design problems leveraging cluster-based mixture models. In forward design, flight behavior is predicted for a given airplane geometry and in reverse design, the geometry is predicted given a target flight behavior. (**b**) Summary of our proposed geometry sampling and optimization using a robot designer. (**c**) Flight behavior distribution and variance for the whole design space of paper airplanes. (**d**) Flight behavior and variance of four sample geometries and their cluster-based behavioral labeling.

Developing a means of solving the forward (geometry to resultant distance or behavior), or the reverse (behavior or distance to geometry) design problem (Fig. 1a), would enable physical programming and design of these structures, with the potential to improving flight capabilities of paper airplanes. Understanding these structures also has intrinsic scientific interest to explain the resulting complex behaviors. In the absence of analytically tractable solutions, physical experimentation is required. Preliminary work explored manually launching paper airplanes and using computer vision to extract flight trajectories^25^. This has examined the lift and drag coefficients for a small number of designs. Resulting from the computer graphics community there is a tool to perform aerodynamics driven optimization to constrain the design space to designs configurations that are likely to actually fly^26^, this is driven by a subset of experimental data from successfully launched airplanes. These approaches which utilize manual experimentation are labor intensive, time-consuming process and are sensitive to changes in fabrication or launching such that the true probabilistic nature of the behavior cannot be explored. To be able to solve the forward and reverse design problem we require large scale, intelligent methods for data-capture and data-driven interpretable models that account for the discrete behavioral groupings and the multiplicitous relationship between geometric parameters and flight behaviors.

The use of robots to perform large scale ‘physical’ experiments for optimization, discovery or modeling is becoming feasible due to increasing versatility of robotic manipulation and improvements in the accessibility of computer vision and optimization methods. The use of robots to automate laboratory tasks for the ‘discovery’ or ‘design’ in chemistry has been shown^27,28^. Closer to the domain of paper airplanes, robotic automation for iterative exploration of falling paper^29^ showed large scale data capture, however, this did not extend to online optimization or model forming. Within the domain of robotics, there has been demonstration of physical realization for testing of soft or deformable systems^30^, which has included using genetic algorithms or Bayesian optimization. Although promising, these approaches do not provide an interpretable model to allow both the forward and reverse design problem to be solved. This has been addressed in other domains for example for the design of biological oscillators^31^ or genetic circuits^32^, but has relied on extensive manual data capture.

We propose a robot designer (Fig. 1b), which fabricates simple paper airplanes, performs experiments, analyzes data, builds models, and optimizes, all without input from humans. Using this approach, over 500 paper airplanes have been fabricated and thrown, with the behavior classified automatically using unsupervised approaches. This sheer number of experiments is far greater than has been seen previously, and shows a repeatability in fabrication and experimentation that allows the true probabilistic and stochastic nature of the flight behavior to be observed. An interpretable Gaussian mixture model (GMM)^33^ is constructed that reflects the cluster-based nature of the design space, where clusters are identified using unsupervised methods in both the high dimensional trajectory data space and geometry space^34^. This model allows for the forward and reverse design problem to be addressed in a probabilistic manner. Furthermore, the GMM-based approach can be used to minimize the design space to allow for efficient and fully automated robot design of paper airplanes for target flight distances.

## Results

The robot designer enables fabrication of a paper airplane constructed from a holder and paper wing planform where the geometry of the wing is parameterized (Fig. 1a). The area and weight of the paper airplanes are fixed, and the holder attachment location is standardized as a function of the wing aerodynamic center (AC). After the robot launches the fabricated airplane, the 2D trajectory of the plane is recorded allowing the distance flown and the characteristics of the trajectory to be analyzed. See Supplementary Video 1 for the robot designer fabricating and launching a paper airplane and *Methods* for more details. Like many physical phenomena, we see discrete behavioral groups of trajectory: nose dive, glide, and recovery glide. The behavior is also probabilistic, with the same design showing different distances traveled and different behaviors with repeated throws. Throughout this work, the three behavioral groups are referred to as ‘Behavior 1’, ‘Behavior 2’, and ‘Behavior 3’ or ‘B1’, ‘B2’, and ‘B3’, respectively. The wings in Behavior 1 follow a trajectory with a curvature, similar to what looks like a ‘nose dive’ which results in a short flight distance. Many wings in Behavior 2 follow trajectories that are more of a ‘glide’ than in Behavior 1 with slightly longer flight distances. In Behavior 3, the flights have a characteristic trajectory containing one or more inflexion point. This is as if the paper airplanes are ‘recovering’ into level flight after a period of nose-down trajectory, often leading to a longer flight distance. See Supplementary Video 2 for example flights showing the three behavioral clusters.

Exploiting the precise and automated nature of the robotic setup, large scale experiments can be performed to enable design optimization. By testing and evaluating many airplanes the design space can be characterized and explored. The data collected can also be used to build a GMM assisted model to predict the probability of a given behavioral type given the geometry. The performance of the GMM assisted model is evaluated by considering the accuracy of the predictions for the forward and reverse design problems. Finally, we demonstrate how developing these models can be used to accelerate real-world robotic optimization of a design—to identify wing shapes that fly a given distance. This section shows key results in the exploration of the design space characteristics and and usage of the probabilistic model. For mathematical details, see *Methods*.

### Exploration of the design space

The design space of paper airplanes is complex, nonlinear and probabilistic—the fluid-solid interactions are challenging to accurately capture making predicting the resultant behavior and flight distance challenging. The robot allows for repeatable and systematic exploration of the design space to capture the relationship between geometric parameters, behavior type, and distance flown. A data-set was created for characterization and development of the unsupervised modelling method by sampling 50 designs across the design space using Latin hypercube sampling^35^. The fabrication and evaluation process takes approximately 4 min for each paper airplane. Each design was fabricated and flown five times, resulting in total 250 flights, to allow the variability in the flight behavior and distance to be captured.

Figure  1c shows a histogram of the 250 flight distances flown by the 50 sample airplanes using 20 bins with a smoothing function fit. The range of distance flow for the three behavioral types is also shown. The airplanes land approximately within 1-$5\, {\textrm m}$, with the distribution skewed toward the lower distances. Thus, the probability of identifying a design to achieve a given target distance varies for different distances. Furthermore, airplane designs from different behavioral groups show different trends in terms of variability. In this sample of wing designs, Behavior 3 shows the largest variability with a range of $2\, {\textrm m}$, and Behavior 1 the least with a range of $1\, {\textrm m}$. Figure  1d shows four examples of wing geometry, with the extracted 2D trajectory for each of the five repeated flights and the variance of the flight distances. The behavior type is labeled automatically using unsupervised clustering on the flight trajectory. Within the five repeated experiments for each design, there can be considerable variation in the distance travelled, and also for the behavioral type. For example, we see for Geometry 2, the same plane design can show a variation of $1.8\, {\textrm m}$. For Geometry 4, its flight trajectory straddles between Behavior 1 and 2. This illustrates the need for a probabilistic model.

**Figure 2:**
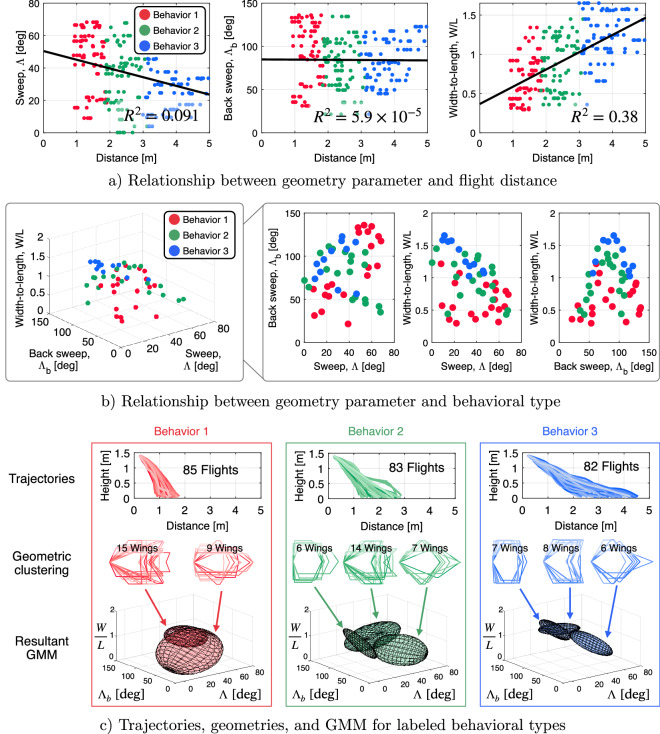
(**a**) Strength of relationship between geometry parameter and flight distance for 250 flights. (**b**) Relationship between geometry parameter and behavior type in geometry space $\mathscr {G}$. (**c**) Flight trajectories, airplane geometries, and GMM representation for labeled behavioral types.

To design airplanes to fly a given distance, an accurate relationship of geometric parameters to flight distance is required. The strength of the relationship between each geometry parameter and distance can be found by plotting the distance for each of the 250 flights against the design parameter and determining the R$^{2}$ value. For the three design parameters we see that this relationship is weak (Fig. 2a), particularly so for back sweep, whereas sweep angle has a weak negative correlation, and width-to-length a weakly positive correlation. The relationships make predicting the distance from geometry challenging.

In addition to analyzing the relationship between geometric parameters and distance we can also explore the relationship between geometric parameters and behavioral type. In Fig. 2b for the 50 airplane geometries, we see groupings of behaviors. However there are overlaps and the behavioral group show a number of distinct sub-clusters which also need to be captured in our approach.

### GMM based modeling

The analysis from the characterization data of 50 airplane designs and 250 flights shows that the behavior is a key means to understand the resulting flight distance, and also for sub-dividing or analyzing the design space.

However, the design space has overlapping clusters that correspond to the different behaviors. Furthermore, the relationship between geometry and behavior is probabilistic, with a given geometry exhibiting multiple behavioral types, and hence resulting in a variability in distance flown. This characteristic demonstrates the need for a *probabilistic* and *cluster-based* approach to analyze the design space, to identify these behavioral groups.

The final form of the model is a collection of Gaussian mixture models, with a single GMM corresponding to a behavior, to indicate the probability of a particular airplane geometry exhibiting each behavior. This GMM assisted model provides an observable and transparent approach to analyzing and representing the behavioral types. Enabling an insightful analysis into the design space, and assist with identifying paper airplanes that fly a given distance.

The GMM assisted model is built from the 50 geometries sampled. All 250 flights are first labeled with a behavior type using the unsupervised clustering method, where the resultant clustered trajectories are shown in Fig. 2c (top). For the three behavioral groups, the trajectories show distinct characteristics. Within these groupings, there are a large number of individual trajectories: 85 for Behavior 1, 83 for Behavior 2, and 82 for Behavior 3. Each of these groups of trajectories contain sub-clusters in the geometry space. To identify these sub-clusters, unsupervised clustering within each behavioral group is performed, using the three geometrical parameters with the number of clusters optimized to find the best clustering.

Within Behavior 1 for example, two distinct clusters of geometries are found, one group with a high *W*/*L*, and a second more trapezoidal in shape. Thus, these two sub-clusters are used to form the GMM, which are represented as the ellipsoids in Fig. 2c (bottom). For Behavior 2, three sub-clusters are found in the geometrical space to form the GMM. The sub-clusters include a group of diamond shaped airplanes (higher $\Lambda$ and lower $\Lambda _{\textrm b}$), rectangular airplanes (higher *W*/*L*), and more triangular shapes (higher $\Lambda$ and higher $\Lambda _{\textrm b}$). Behavior 3 is also formed from three groups—the characteristics are similar to those in Behavior 2, but the geometries are grouped much stronger even by visual inspection. Comparing the GMMs developed for the three behaviors, it can be seen there is overlap, reflecting the probabilistic nature, and also that the behaviors span different volumes of the design space, with some of them more tightly clustered than the other.

### Behavioral prediction and programming

The GMM for each behavior built using the data collected by the robot can be utilised in two ways. First, to solve the so-called forward design problem: predicting the probability of behaviors for a given geometry. Predicting the behavioral probability can be used to provide an estimation of the average and distribution of likely distances flown. The second assessment, is to use the model to perform the reverse design problem: identifying designs that are likely to have a given probability of certain behaviors. The interpretability of the GMM is advantageous for performing this reverse design method over more traditional learning methods.

To assess the performance of the model for the forward design problem, GMMs were formed from 94 sampled geometries, each with five flights. $16\%$ of the data was withheld for evaluation of the model. After forming the GMMs for each behavior type, the probability of a given behavior for a specific geometry can be determined by integrating the probability distribution function from the GMM.

**Figure 3:**
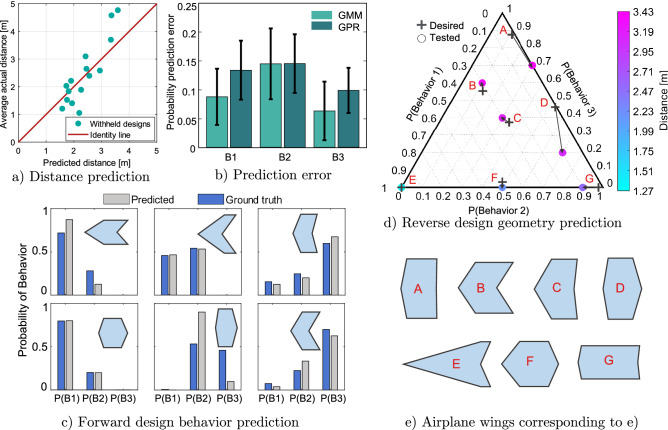
(**a**) Comparison of average actual distance from experimentation to the predicted distance for airplane designs withheld from the model. (**b**) Comparison of absolute average error of probability prediction for all withheld airplane designs between the GMM-assisted method and GPR. (**c**) Forward design behavioral probability prediction of a sample of wings held out from the model compared to its ‘ground truth’ behaviors. (**d**) Reverse design behavior prediction for chosen target probabilities with corresponding average flight distances. (**e**) Airplane geometries that are predicted to display the target behavioral probabilities in reverse design.

Figure 3c shows the ground truth and predicted probability of behavior for a selection of wing geometries held out from the model. The probabilities are generally in agreement between the ground truth and predicted. It can be seen that certain wings are predicted to be very distinctly Behavior 1 while for example, certain wings are close to being half-way between two behaviors. In general, wings that fall in Behavior 1 fly very similarly however many times they are flown. The same is true for many wings that fall in Behavior 3. However as Behavior 2 is in the middle range, airplanes that display this behavior have characteristics overlapping with the other two behaviors.

The average absolute error across all withheld geometries is shown in Fig. 3b for each behavior type performed through the GMM-assisted method and Gaussian process regression (GPR). It can be seen that for all behavioral types the probability prediction errors are lower with the GMM-assisted method compared to GPR. Finally the probability predictions can be used to accurately predict the flight distance for a given geometry. As shown in Fig. 3a, for most of the withheld geometries, the flight distances can be predicted within 30% accuracy using the behavioral probability predictions compared to the measured distances. The forward design results show that the cluster-based mixture model is able to capture the probabilistic nature of this complex system to enable accurate predictions of behavior type and also estimate the distance flown.

To assess the performance of the GMM assisted model for the reverse design problem, the model is used to predict wing designs that give rise to a specific probability of behavior. To explore this problem, we pick 7 different desired target combinations of probability of behaviors. Combinations of probabilities were chosen that spanned the range of achievable outcomes. For the predicted geometry, each of these seven paper airplanes are flown 10 times each and the trajectories labeled using unsupervised behavioral clustering to allow the probability of the different behaviors to be determined. The desired and measured probabilities are shown on the ternary plot (Fig. 3d) with the average distance also represented. The corresponding predicted geometries are shown in Fig. 3e. For five of the airplane geometries (B, C, E, F and G), the error is less than 5%. However for geometries A and D where the probability of Behavior 1 is 0%, the airplanes show a larger error of more than 10%, with the airplanes showing lower than expected probability of Behavior 3. As seen in the behavioral distribution and flight distance variance of the whole design space of the paper airplanes, geometries labeled as Behavior 1 tend to follow very similar, short-distance trajectories—having no or very little probability of Behavior 1 makes a flight more unpredictable. Furthermore, we also saw the skew in the distribution of flight distances toward shorter flights, and a high variance of trajectories labeled as Behavior 3. These also contribute to the result and explain the larger error for the geometries A and D. In this way, the reverse design results show that it is possible to program probabilistic behaviors in the design of a paper airplane wing.

### Bayesian optimization for target distance

**Figure 4:**
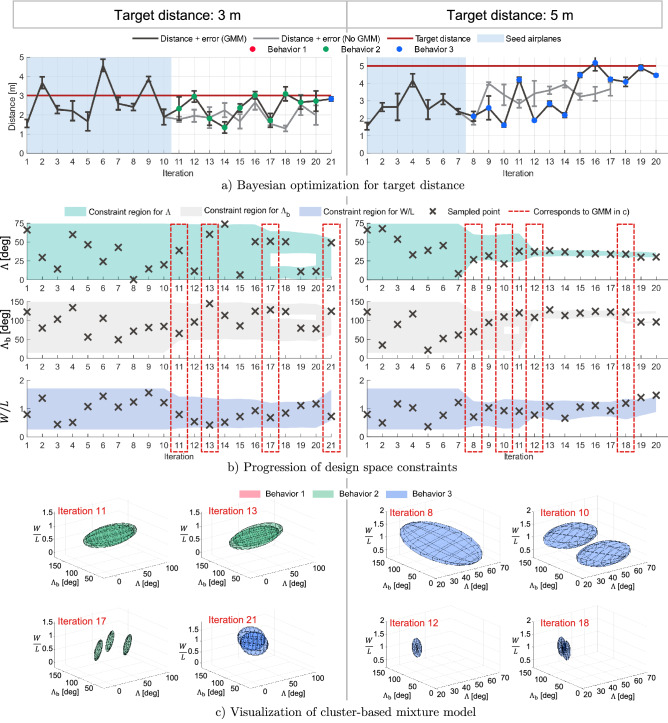
Experiment results of iteratively designing a paper airplane to fly a target distance of $3\, {\textrm m}$ (**left**) and $5\, {\textrm m}$ (**right**). (**a**) GMM-assisted and unassisted Bayesian optimization results of flight distance at each iteration with dots indicating the behavioral cluster the optimizer searches in. (**b**) Visualization of the geometry space constraints at each iteration and the sampled points. (**c**) Visualization of the geometric search space created by the GMM at the indicated iteration number, also corresponding to the dashed red box in (**b**).

The GMM can be leveraged in conjunction with the automated robotic system to enable iterative, directed search to achieve a wing geometry that has a high likelihood of achieving a target flight distance. Bayesian optimization is used to perform iterative search of the design space, with each wing geometry tested five times by the robotic setup. Bayesian optimization is useful in trying to capture the stochastic and probabilistic nature of this design problem and allows for a sequential decision making, iteratively sampling new geometries^36–40^. At each iteration of the optimization algorithm, the GMMs for all behaviors are updated. From this, the design space for the Bayesian optimization is adapted online to be defined by the GMM which has an average flight distance which is closer to the target distance. To explore the improved performance of this GMM-assisted method, Bayesian optimization for two target distances, $3\, {\textrm m}$ (Fig. 4 left) and $5\, {\textrm m}$ (Fig. 4 right), are conducted. For each target distance the experiment was repeated twice, one where the optimization was aided by the GMM and one where Bayesian optimization was solely used.

The GMM-assisted optimization must be initialized by generating a behavioral model. The flight performance data of 10 wing geometries, randomly selected from the Latin hypercube samples as ‘seeds’ were used to create the cluster-based GMM. A target of $3\, {\textrm m}$ was chosen because none of the seed airplanes landed within a $20\, {\textrm cm}$ tolerance of $3\, {\textrm m}$, thereby challenging the optimization to search for wing geometries not yet explored in the seeds. As shown in Fig. 4a, the model-assisted experiment reaches within $5\, {\textrm cm}$ of the target at iterations 12 and 16. However, since the target is an intermediate distance not at the extremes of the system, there are multiple solutions or geometries that could potentially reach this target—the experiment progression also shows that the optimizer is still exploring possible designs (Supplementary Fig. S6). At iteration 18 and onward, the target of $3\, {\textrm m}$ is reached consistently within a small tolerance and the ellipsoids (Fig. 4c) of allowable geometries also show how the space in which the experiment explores is being constrained (Fig. 4b). On the other hand the standard Bayesian optimization is never able to find a solution that is closer than $30\, {\textrm cm}$ within the target.

To further validate the model-assisted optimization method, we now reduce the number of seed airplanes as well as set a target distance of $5\, {\textrm m}$ that from prior design space exploration we know to be at the extreme of this system. Contrasting from the previous experiment with a target distance of $3\, {\textrm m}$, only a few wing geometries are expected to meet the new target. The aim of this experiment was therefore to see how quickly the model-assisted optimization would converge to a solution compared to the standard Bayesian optimization. By iteration 12, the geometry space is well-constrained (Fig. 4, Supplementary Fig. S6). The model-assisted method successfully reaches the target within a $20\, {\textrm cm}$ tolerance at iteration 16 and quickly again at iteration 19. On the other hand, the standard optimization is stuck at a local maximum where the maximum obtained distance is $20\%$ lower than the target. These results demonstrate that by introducing a probabilistic cluster-based model to constrain the design space, this can be used to reach a target faster in an optimization problem.

## Discussion

The robot designer we propose can advance our understanding and exploration of design problems that may be highly probabilistic, and could otherwise be challenging to observe any trends. In this work, we present our approach in creating a cluster-based mixture model to perform behavioral labeling to model the relationship between the airplane geometry and its flight behavior. In forward design, our GMM-assisted model predicts the behavioral probability with an error of less than 15% for unseen airplane geometries. In reverse design, the behavioral probability for many of the predicted airplane geometries are less than 5%. The model is also shown to better inform the sampling process of a Bayesian optimization process to reach a particular target flight distance in fewer iterations. The GMM-assisted optimization for the longer target distance of $5\, {\textrm m}$ reaches the target in 9 iterations after the seeds. The wing geometries that fly far have high *W*/*L* and low to mid $\Lambda$. This is quite visually different from the planform of a classic dart paper airplane^21^, perhaps a shape often regarded as ‘high performing’ by a human designer.

It was found through automated robotic experimentation and unsupervised clustering that the system of paper airplanes exhibit three distinct clusters of behavior: nose dive, glide, and recovery glide. Their behaviors are not only characterized by the flight distance but also by the flight trajectory. The probabilistic nature of the problem means multiple geometries exhibit similar behaviors, while concurrently a single geometry could exhibit multiple behaviors. This characteristic demonstrates the need for a probabilistic and cluster-based approach to analyze the design space. The model we use together with the robotic setup uses Gaussian mixture models to build a cluster-based probabilistic model to map geometry parameters to behavioral groups. Compared to other function approximation methods, this GMM-based model outperformed in its performance in forward and reverse design, while maintaining a good interpretability of its inner workings.

This approach could be improved and further validated by varying the airplane geometry parameterization, fabrication method, initial conditions, and selection/tuning of the optimization method. The chosen airplane geometry allows for simple ‘conventional’ wing shapes using only three parameters. The definition of the parameters and/or the number of parameters can be varied. Fabrication methods, for instance, such as the paper material is an interesting factor to explore further. A more flexible material may induce increased stochastic behavior, while a stiffer material (given that other factors such as wing area are tuned accordingly) may lead to reduced stochasticity and/or increased flight distance. Using alternate fastening methods for the holder may lead to weight changes that would change the probabilistic landscape of the system. Another factor that this work would benefit by investigating further is the launching conditions, such as the launching mechanism, velocity of the robot arm pushing the paper airplane, and the pitching of the nose at launch. Currently, these variables are arbitrarily chosen to ensure the repeatability of each launch while successfully accelerating the paper airplane. Small variation in the arm velocity for example, most likely will not change the airplane behaviors. Yet, large changes of arm velocity may not function mechanically with the current launch mechanism—the success of the launch depends on the consideration of the entire setup as a whole. While there are many fabrication and setup factors that can be considered, our probabilistic approach remains workable for the forward and reverse design problems of paper airplanes. To conclude the remarks for future recommendations, in this work, two target flight distances were chosen to optimize the airplane geometry using Bayesian optimization assisted by the GMMs. This optimization can be improved by searching for other flight distances, or other metrics such as flight time, velocity, or centerline deviation. The optimization algorithm itself can be modified by searching for better parameters of the Bayesian exploration or using an alternative sampling method.

It is demonstrated in this work that the airplane flight trajectory behaviors are complex, where the mapping between the geometry and behavior is unintuitive, probabilistic in nature, and cannot be solved analytically. Our approach serves as an exploration in using a robotic designer for understanding and exploiting a complex physical system. In the future, this approach could be extended to increasingly complex design spaces or other robotic setups, such as the design of MAVs to optimize its wing shape to maximize flight distances.

## Methods

**Figure 5:**
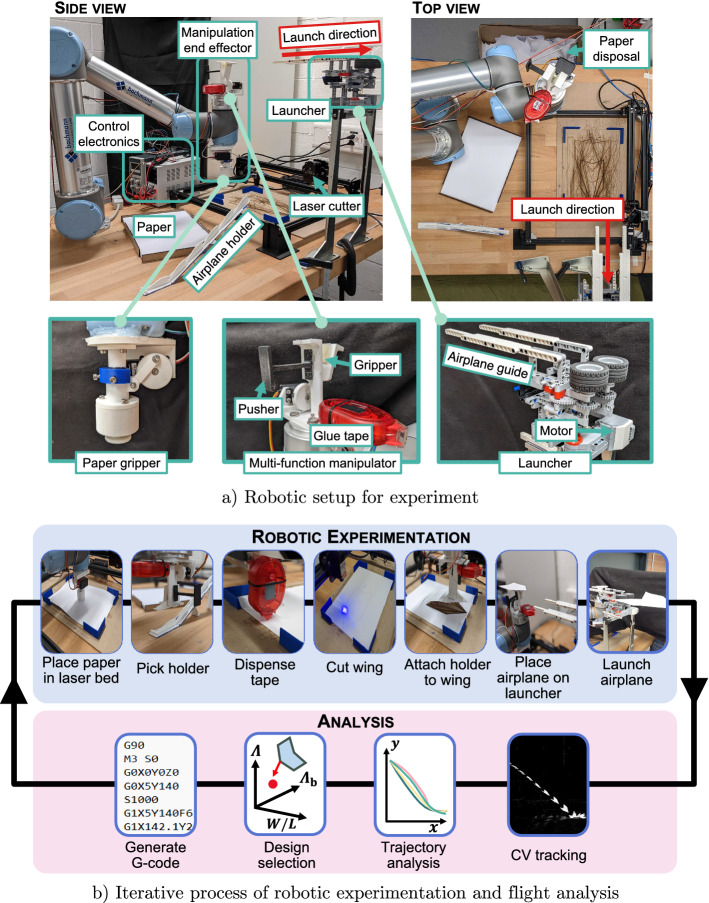
(**a**) Robotic experimental setup for paper airplane fabrication and launching. (**b**) Iterative process followed by the robot designer alternating between experimentation and analysis in a closed-loop.

Central to the work is the development of methods for automating the fabrication, analysis, and model generation to enable design optimization.

### Parametric wing design

The paper airplane is composed of a wing planform cut out from deformable paper which is glued to a 3D-printed holder as shown in Supplementary Fig. S1 (right). The holder acts as a weight for the paper airplane structure to control the pitching of the nose and also as a structure through which the robot can manipulate the airplane. The parameterized paper airplane wing is shown in Fig. 1a and Supplementary Fig. S1 (left). The wings are defined using three geometric parameters: front sweep angle, $\Lambda \in \left[ 0^\circ {\textrm ,}\,74^\circ \right]$, back sweep angle, $\Lambda _{\textrm b} \in \left[ 14^\circ {\textrm ,}\,152^\circ \right]$, and width-to-length ratio, $W/L \in \left[ 0.25{\textrm ,}\,1.71\right]$. The 3-dimensional geometric design space could be described in many different ways—the silhouette score is used to find the geometric parameters that best cluster the different behavioral groups. Sample wings are shown in Supplementary Fig. S2. Several design constraints were placed to facilitate and standardize the automatic design of wings: i) wing surface area is fixed at $200\, \mathrm {cm^2}$; ii) number of vertices is fixed at six; iii) wing must fit on an A4-sized paper with a $5\, {\textrm mm}$ margin all around; iv) root chord, $c_{\textrm r}$ must be larger than tip chord, $c_{\textrm t}$; v) tip chord must be greater than or equal to zero; vi) the airplane nose must coincide with the $(5,105)\, {\textrm mm}$ point as shown in Supplementary Fig. S1 (center). Fixing the wing area, paper material, and glue application, and thereby also fixing the total mass at $6\, {\textrm g}$ allows meaningful comparison of different paper airplanes by keeping the wing loading constant. The fabrication of the wing is further standardized by always attaching the center of mass of the holder at a point on the wing that is 30% of length, *l* in Supplementary Fig. S1 (center) forward starting from the aerodynamic center (AC)^41^.

### Hardware setup

A robot setup (Fig. 5a) has been created to fully automate the fabrication, testing and data collection of paper airplanes. The key components of the setup include a UR5 robot arm to perform the necessary manipulation tasks, a desktop laser engraver to cut the wing planform from paper and an airplane launching system. The UR5 is equipped with two grippers: a ‘tack’ based gripper with an actuated ring for releasing objects that can be used to move paper around the setup, and a two finger gripper with silicone padding for manipulating the airplane holder and the assembled airplane. A glue tape dispenser is fixed to this gripper to enable the airplane holder to be attached to the cut wing shape.

A desktop laser engraver is used to cut the wing shape from paper enabling a precise and repeatable cutting process. The engraver utilizes G-code for coordinate specification and cutting parameters. G-code is automatically generated based on a wing geometry that has been determined. The paper from which the wing is cut out needs to be resupplied by a human after fabricating approximately 100 paper airplanes. The launcher system is placed $1.4\, {\textrm m}$ above the ground and uses two high friction rotating wheels at then end of guide rails. When the airplane is pushed into the high speed wheels the holder is caught by the wheels and is rapidly accelerated to a fixed velocity launching the paper airplane.

The robotic setup is in an enclosed room of approximate size $5\times 8\, {\textrm m}$ to minimize the effects of drafts and external disturbances. Cameras are placed to capture the 3D trajectory of the airplane, with one perpendicular to the plane of the trajectory, the other with a viewpoint along the trajectory. The system diagram of the experimental setup is shown in Supplementary Fig. S3.

### Robotic fabrication process

The robotic fabrication process of a paper airplane is summarized in Fig. 5b. Once a wing geometry for testing is selected, the robotic setup is able to fabricate the paper airplane and launch it. The fabrication process begins with the robot arm picking a single sheet of A4-sized $160 \, \mathrm {g/m^2}$ DCP paper and placing it on the laser engraver bed. Then the robot picks a 3D-printed holder from a rack of holders. Glue tape is then applied to the sheet of paper, at a location which is programmatically chosen based on the aerodynamic center of each wing. Glue tape ensures that there is minimal weight increase on the paper airplane and that the adhesive properties are strong enough for the chosen paper and the holder material. After glue application, the laser engraver cuts out the wing shape. When the cutting process is completed, the holder is attached to the wing using the previously dispensed glue tape. The gripper grasps the assembled paper airplane, places it on the guide rails of the launcher, and finally performs a launch sequence by pushing the paper airplane at an acceleration of $1\,\mathrm {m/s^2}$ to the launcher’s wheels rotating at 160 RPM. The guiding rails mounted horizontally parallel to the ground, and there is no pitching angle of the launched airplane. Flight data is acquired using two cameras and automatically analyzed. During the flight sequence and subsequent data analysis, the robot picks the waste spare paper on the laser engraver bed and disposes of it. To enable robust and reliable fabrication, the robot arm is controlled using force control when performing tasks such as picking up paper or the flight holder where the height of the position of the component can be variable.

### Data capture

The paper airplane flights are recorded using two Logitech cameras recording at $1280\times 720$ resolution at $30\, {\textrm FPS}$. A visualization of the data capture setup from the top is simplified and illustrated in Supplementary Fig. S4. The first camera, placed perpendicular to the plane of flight, is used to obtain the 2D trajectory of the flight from the side. A second camera is placed looking along the flight path to capture the out of plane motion to allow the flight distance to be adjusted for this motion. To extract the trajectory from the raw videos, each frame is compared to the first frame to obtain a binary difference image. Each of these images is blurred, and a binary mask is created to detect the largest object in each frame, which corresponds to the paper airplane. The mean location of the object in the two dimensions of the image frames is used to identify the location of the airplane in each frame. The final $3\, {\textrm cm}$ before the airplane touches the ground is discarded as the trajectory and data capture is affected by the ground. A high friction mat covers the landing region of the paper airplane so that they slide on the floor minimally after first touching the ground.

To correct for any out of plane motion in the trajectory extracted from the side camera, the final landing spot is extracted from the motion in the second camera and this is used to scale the trajectory to the true distance accordingly. The pixel coordinates are converted into world coordinates of the environment using 2D projective transformation^42^ from vision markers placed on the floor. This results in a trajectory which is defined by a series of points in 2D space.

For each trajectory it is necessary to determine the distance flown and also the behavior label of each trajectory. To do this, it must be converted into a usable form. A polynomial fit is used as it is well suited to the form of the trajectories and also has the necessary information in a reduced form to enable labeling of the behavior. A 5th degree polynomial is able to capture most trajectories well. As all flights fall approximately as a downward sloping trajectory, the 0th and 1st coefficients are discarded. Each flight can therefore be described in terms of the flight distance, *s* and the 2nd to 5th degree polynomial coefficients, $c_1$, $c_2$, $c_3$, and $c_4$, respectively.

**Figure 6:**
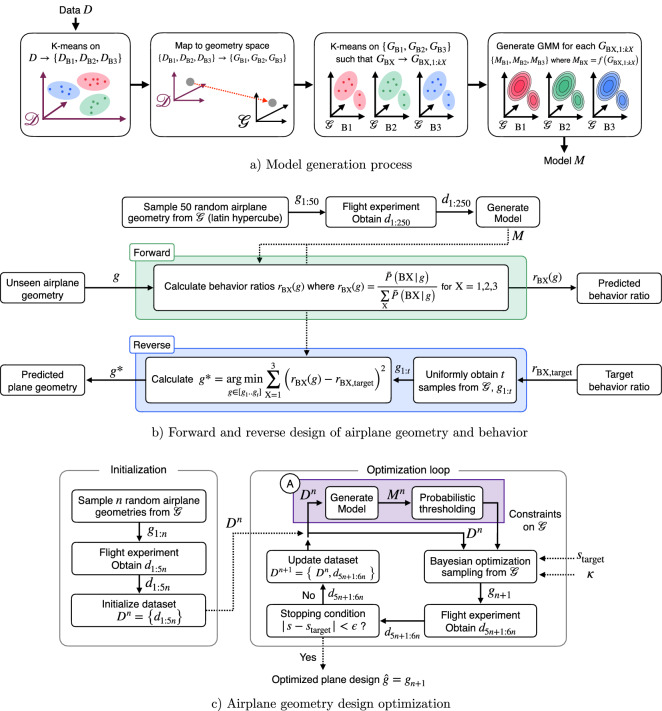
Schematic of the proposed method for (**a**) generating the GMMs for each behavioral type; (**b**) forward and reverse design problem in mapping between airplane geometry and behavior; (**c**) GMM-assisted and unassisted Bayesian optimization of airplane geometry.

### Data-set generation

To understand the design space of the system and ultimately generate the GMM-assisted model, 50 representative airplane geometries are sampled. To begin the process, first 800 airplanes which *satisfy the constraints* mentioned in “Parametric wing design” are uniformly sampled. Then, to choose a representative sample of 50 airplane geometries, Latin hypercube sampling (LHS) is used to sample 50 airplane geometries which *do not necessarily satisfy the constraints* mentioned in “Parametric wing design”. Finally, for each airplane geometry obtained through LHS, a corresponding airplane geometry from the initial 800 geometries is found which minimizes the RMSE of the parameters, resulting in 50 airplane geometries which *satisfy* the constraints.

### Creating a cluster-based behavior model

We observe that the system of paper airplanes form behavioral clusters within the geometric space (Fig. 2b). The flight trajectories and distances shown in Fig. 1c,d also call for a method that is able to capture their probabilistic nature of behaviors. To obtain an insight into their behavior, a cluster-based Gaussian mixture model (GMM) assisted method is chosen instead of methods utilizing neural networks, for example, so the mapping between the geometry and behavior are interpretable to allow for solving the forward and reverse design problems.

The process of creating the model is shown in Fig. 6a. The model requires an aggregate data-set $D=\left\{ d_1,d_2,\ldots \right\}$, where each data point $d \in \mathscr {D} \subseteq \mathbb {R}^5$, represents the behavior of each flight (as in $d=\left[ s,c_1,c_2,c_3,c_4\right]$ from “Data capture”). Each behavioral type must first be automatically labeled, such that the data corresponding to each behavior can be used to form a GMM. To do so, unsupervised *k*-means clustering is performed on *D* with $k=3$. Here, $k=3$ was chosen by calculating the Calinski-Harabasz criterion^43^ for each $k \le 6$ using only the polynomial coefficient information of *D*. $k=3$ matches the number of trajectory types visually observed (Fig. 1a) and provides confidence in the results of unsupervised clustering. This calculation is performed once, and $k=3$ will be used for all future model generation steps.

With this clustering, each flight data *d* is classified into one of three behaviors (B1, B2, B3). this results in a behavior-classified data-set $D = \left\{ D_{\textrm B1},D_{\textrm B2},D_{\textrm B3}\right\}$ and a data-set of a behavior *X* becomes $D_{{\textrm B}X}=\left\{ d_{{\textrm B}X,1},d_{{\textrm B}X,2},\ldots \right\}.$

Each of the data points *d* in the set *D* can evidently be mapped to a wing geometry $g=\left[ \Lambda ,\Lambda _{\textrm b},\frac{W}{L}\right]$ in the geometry space $\mathscr {G} \in \mathscr {G} \subseteq \mathbb {R}^3$. When we map every behavior-classified data point from $\mathscr {D}$ to $\mathscr {G}$, we result in a behavior-classified geometry set $G = \left\{ G_{\textrm B1},G_{\textrm B2},G_{\textrm B3}\right\}$.

The automatic classification allows for distinction between large differences in behavior. However, each $G_{{\textrm B}X}$ spans are large space in $\mathscr {G}$, and lacks specificity. For instance, the R$^{2}$ values for each geometric parameter in relation to the flight distances are still weak (Supplementary Table S1). Therefore, each behavior geometry set $G_{{\textrm B}X}$ is further clustered using *k*-means, this time in geometry space $\mathscr {G}$. The number of clusters is not chosen a priori, and its value for each geometry set $G_{{\textrm B}X}$ is automatically determined by Eq. (1) by evaluating the silhouette score.

1 $$\begin{aligned} kX=\mathop {{\textrm argmax}}\limits _{kX}\ {\textrm silhouette}\left( k{\text{- }}{\textrm means}\left( G_{{\textrm B}X}\right) \right) \end{aligned}$$

As a result of this secondary clustering process, each point in $\mathscr {G}$ has a classification of its behavior, ${{\textrm B}X}$ and the sub-cluster ‘component’ *y*: $g_{{\textrm B}X,y}$.

Finally, for each behavior classification, a Gaussian mixture model is formed using the sub-clustering. The mixture model, $M_{{\textrm B}X}$ is generated for each geometry set $G_{{\textrm B}X,1:kX}$ such that $M=\left\{ M_{\textrm B1},M_{\textrm B2},M_{\textrm B3}\right\}$. Since the geometry space $\mathscr {G} \subseteq \mathbb {R}^3$, the resulting model is a multivariate Gaussian mixture model with $k{\textrm X}$ components that vary for each behavior. The set of cluster-based mixture models *M* serves as a function to relate the airplane geometry and its probabilistic flight behavior.

### Using the cluster-based behavior model

The cluster-based model can be used to map between the wing geometry and probability of each behavior of the paper airplane, where the process is shown in Fig. 6b. In forward design, the probabilistic behavior can be predicted for an unseen airplane geometry. In reverse design, the geometry of the airplane can be predicted for a specified probabilistic behavior.

#### Forward design: geometry to behavior

Before performing the forward design process of predicting behavior for a specified geometry, the model *M* must be generated. *t* paper airplane geometries are first sampled from $\mathscr {G}$ using Latin hypercube sampling. Each airplane geometry $g_{1:t}$ are flown five times each to obtain the data points $d_{1:5t}$. *M* is then generated using $d_{1:5t}$ via the method described in “Creating a cluster-based behavior model”.

Using the cluster-based mixture model, we wish to determine the probability of a particular airplane geometry, *g* exhibiting a behavior, ${{\textrm B}X}$, $P({{\textrm B}X}|g)$. To simplify the computation, we consider an approximate solution, as shown in Eq. (2).

2 $$\begin{aligned} \begin{aligned}{}&P({{\textrm B}X}|g) \approx \bar{P}({{\textrm B}X}|g) =\max _{i\in [1\ldots kX]} \int ^\infty _{-\infty } h_{{\textrm B}X,i}(x) \mathbbm {1}(x, g)dx \quad x, g \in \mathscr {G}\\&\text {Where} \ h_{{\textrm B}X,i}(x) = \frac{1}{\sqrt{(2\pi )^3|\varvec{\Sigma }_{\textrm BX, i}|}} \exp \left( -\frac{1}{2}({x}-\mu _{{\textrm B}X,i})^T{\varvec{\Sigma }_{{\textrm B}X,i}}^{-1}({x}-\mu _{{\textrm B}X,i}) \right) \\&\quad \text {is the }ith\text { mixture component of behavior }{{\textrm B}X},\text { and} \\&\mathbbm {1}(x, g) = {\left\{ \begin{array}{ll} 1,&{} \text {if } h_{{\textrm B}X,i}(x) \le h_{{\textrm B}X,i}(g)\\ 0, &{} \text {otherwise} \end{array}\right. } \\&\quad \text { is the indicator function to define the region of integration.} \end{aligned} \end{aligned}$$

The integral in Eq. (2) is computed approximately through a discrete summation shown in Eq. (3). The *N*’s represent the minimum and maximum bounds of the numerical integration for the three geometric parameters centered around the mean of each in the GMMs.

3 $$\begin{aligned} \begin{aligned} \sum _{x_1 = -N_1}^{N_1}\sum _{x_2 = -N_2}^{N_2}\sum _{x_3 = -N_3}^{N_3}\ h_{{\textrm B}X,i}(x) \mathbbm {1}(x, g)\Delta x_1\Delta x_2\Delta x_3 \quad \text {where} \ x = [x_1, x_2, x_3] \end{aligned} \end{aligned}$$

Next, the behavior ratios, $r_{{\textrm B}X}(g)$ of a behavior for a given wing geometry can be calculated as Eq. (4). The $r_{{\textrm B}X}(g)$ is a vector of three probability values from 0 to 1 that represents how likely it is for a wing geometry, *g* to exhibit a behavior (B1, B2, B3). In the same way, the probabilistic behavior of an ‘unseen’ wing geometry held out during the model generation, can be predicted.

4 $$\begin{aligned} r_{{\textrm B}X}(g)=\frac{\bar{P}\left( {\textrm B}X|g\right) }{\sum \limits _{X}\bar{P}\left( {\textrm B}X|g\right) }\text {, where } X=1,2,3 \end{aligned}$$

#### Reverse design: behavior to geometry

The generated model can also be used for reverse design where a target behavior can be mapped backed to a wing geometry. The target behavior ratio, $r_{{\textrm B}X,{\textrm target}}$ is compared to the behavior ratio, $r_{{\textrm B}X}(g)$ for $g_{1:t}$ geometries that was used to generate the model. The RMSE is calculated between the two ratios and the predicted airplane geometry, $g^*$ is chosen using Eq. (5). To verify the accuracy of the reverse design problem, the geometries identified as $g^*$ were flown ten times each and their behavioral types identified as shown previously in “Behavioral prediction and programming”.

5 $$\begin{aligned} g^*=\mathop {{\textrm argmin}}\limits _{g \in \left[ g_1,\ldots g_t\right] }\sqrt{\frac{1}{3}\sum _{X=1}^{3}\left( r_{{\textrm B}X}(g)-r_{{\textrm B}X,{\textrm target}}\right) ^2} \end{aligned}$$

### Optimization for distance

The behavioral model can also be used in an iterative optimization loop, where the aim is to search for an airplane geometry which reaches some objective function. At each optimization iteration, the behavioral model can be used to constrain the search space by identifying regions of interest based on the objective function. This method of utilizing the behavioral model is compared against the same optimization process without this additional constraint provided by the GMMs.

In this work, the objective of the optimization is a target flight distance. Bayesian optimization^44^ is used to perform the selection of airplane geometries at each iteration. The optimization method is described visually in Fig. 6c, and the purple shaded box highlights where the behavioral model is used in the process.

To initialize the optimization process, *n* random airplane geometries are sampled from $\mathscr {G}$ uniformly. These $g_{1:n}$ paper airplanes are flown five times each to produce $d_{1:5n}$ flight data points, which becomes the initialization data-set, $D^{n}=\left\{ d_{1:5n}\right\}$ (where the superscript denotes the optimization iteration count).

The model is used to constrain the search space of the Bayesian optimization process. At every iteration, the model $M^n$ is generated using all of the data captured including the initialization process.

Then, one out of three GMMs, $M_\mathrm {B^*}$, is selected, where $\mathrm {B^*}$ is the behavior which minimises the difference of the median of the flight distances in each behavior data-set and the target distance. Using Eqs. (2,3), the set of all points $\mathscr {C} \subseteq \mathscr {G}$ where $\bar{P}({\mathrm {B^*}}|g) > 0.7$. The Bayesian optimization sampling process will only search within $\mathscr {C}$ to output the design $g_{n+1}$.

The newly sampled geometry, $g_{n+1}$ is flown five times to obtain the next data points, $d^{n+1}=d_{5(n+1)-4:5(n+1)}$. The aggregate data-set is updated with the new five data points as in $D^{n+1}=\left\{ D^n,d^{n+1}\right\}$. In this way, the updated behavioral model becomes $M^{n+1}$ in the next iteration. The optimization is continued until a predetermined stopping criterion is met to produce the optimized airplane design, $\hat{g}=g_{n+1}$.

In the standard Bayesian optimization, the *n* airplanes used to create the model are used as ‘seed’ airplanes, and the behavioral model and the geometric space constraining is not performed.

## Supplementary Information

Supplementary Information 1.

Supplementary Video 1.

Supplementary Video 2.

## Data Availability

The associated code and data is available here: https://gitlab.epfl.ch/obayashi/paper-airplane
