# Supplementary material for: Robotic automation and unsupervised cluster assisted modeling for solving the forward and reverse design problem of paper airplanes
Source: Sci Rep. 2023 Mar 14;13:4212. doi: 10.1038/s41598-023-31395-0 (PMC10015042; doi:10.1038/s41598-023-31395-0)
Supplement: Supplementary file 1 — Supplementary Information 1. [file 41598_2023_31395_MOESM1_ESM.pdf]

Robotic Automation and Unsupervised Cluster Assisted Modeling for Solving the Forward and Reverse Design Problem of Paper Airplanes: Supplementary Material

Nana Obayashi, Kai Junge, Stefan Ilić, & Josie Hughes

Supplementary Figures

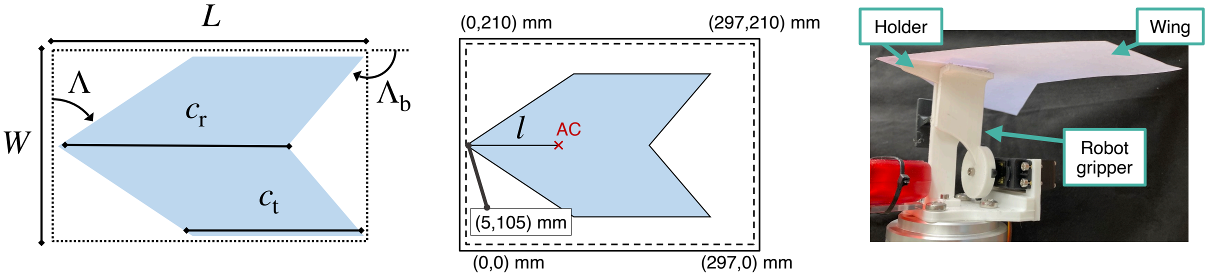

Figure S1. Left: Parameterization of a paper airplane wing. **Center:** Placement of a paper airplane wing geometry on an A4-sized paper. **Right:** Fabricated paper airplane held by the robot about to be placed on the launcher.

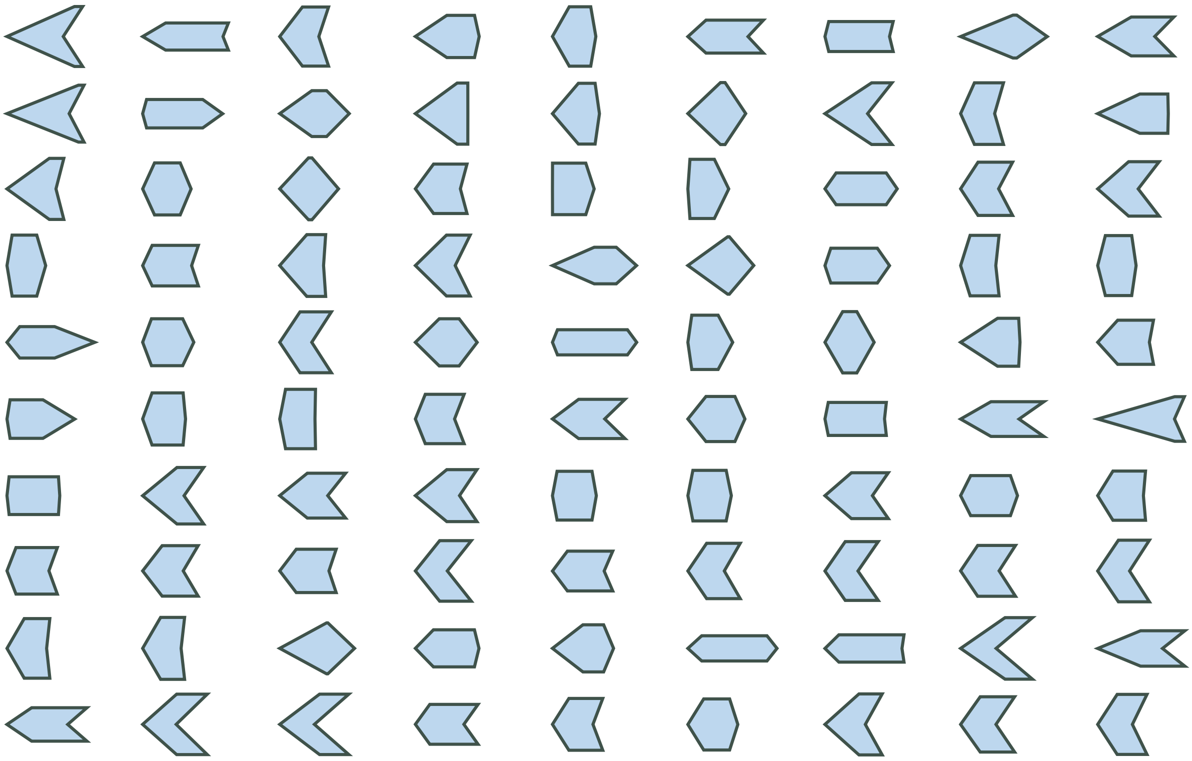

Figure S2. Selection of paper airplane wing geometries used in this work.

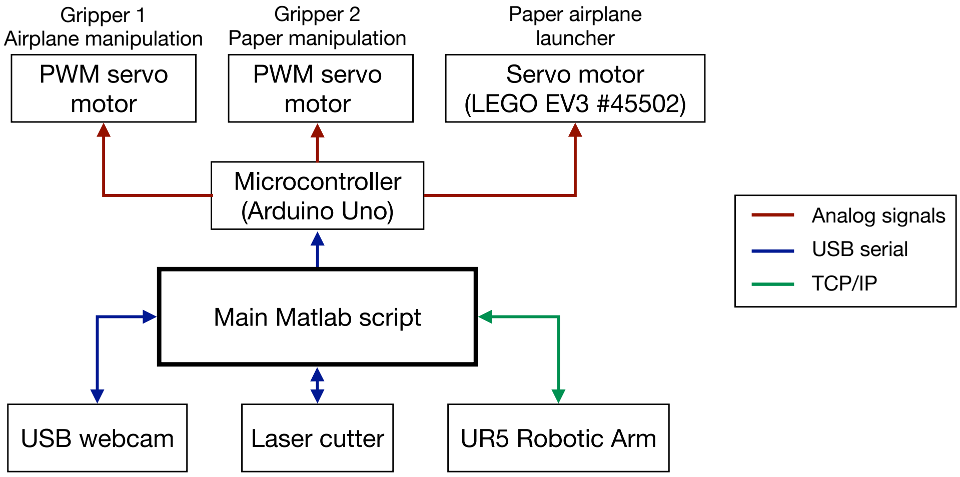

Figure S3. System diagram for robotic setup.

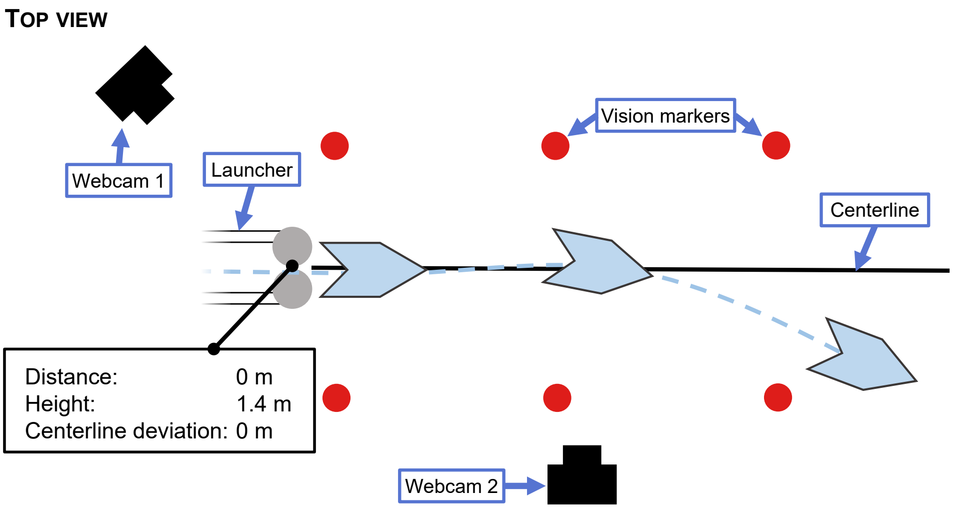

Figure S4. Top view of the data capture setup and the room where flight experiments are conducted.

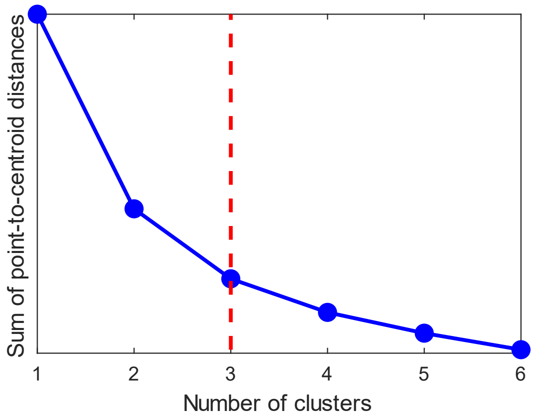

Figure S5. Sums of point-to-centroid distances for varying number of clusters of airplane behaviors.

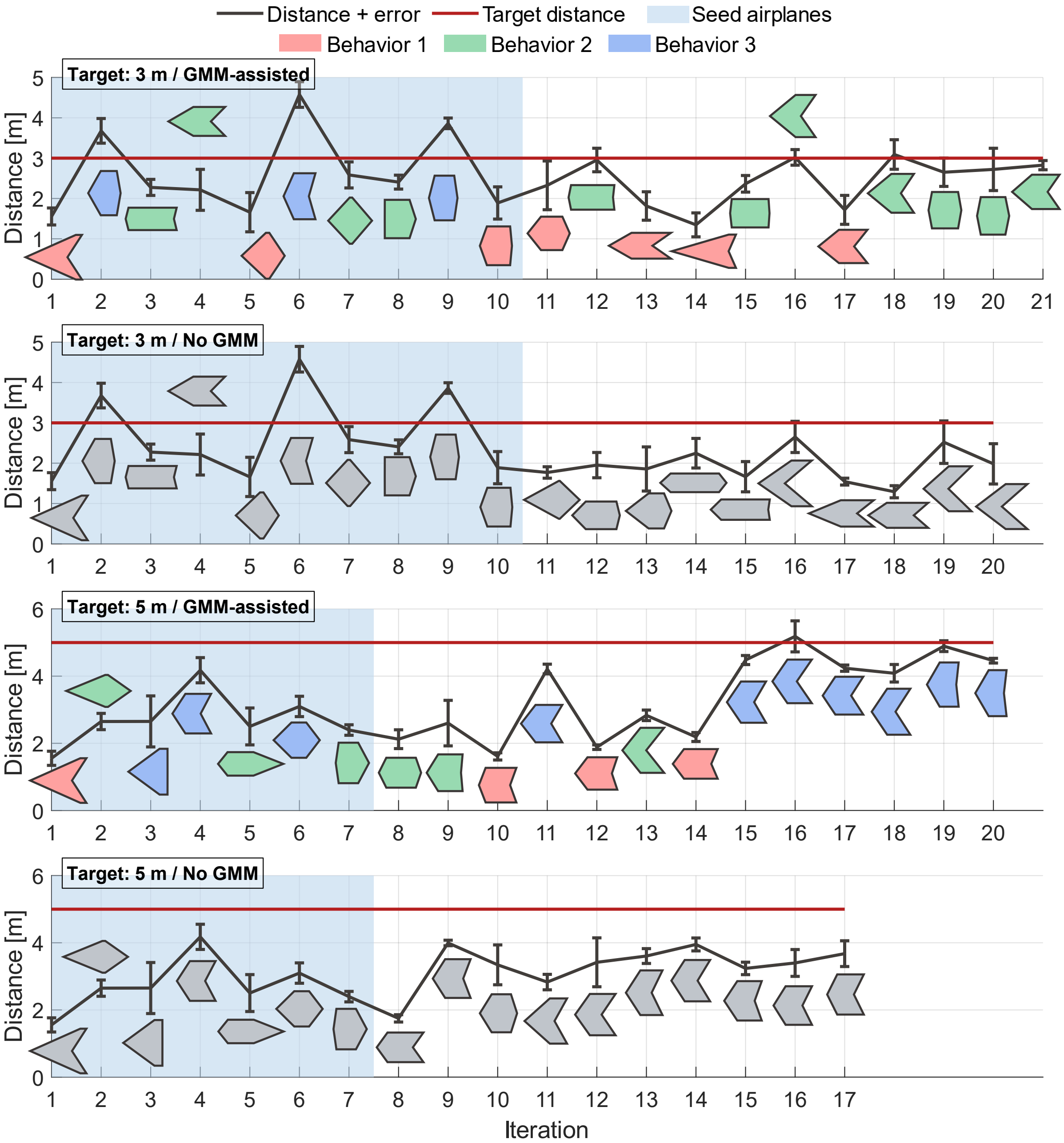

Figure S6. Flight experiments from Fig. 4 showing the airplane wing that was tested to obtain the corresponding distances. The wings are colored according to the behavioral label determined by unsupervised clustering.

	R^2 values		
	**B1**	**B2**	**B3**
**Sweep, Λ [deg]**	0.007	0.015	0.004
**Back sweep, Λ_b [deg]**	0.004	0.001	0.145
**Width-to-length, W/L**	0.162	0.033	0.031

Table S1. R^2 values of the relationship between the geometric parameters and flight distance for each behavioral type.

Supplementary Description

S.1. Experimental Details

The wing planform can be described by several parameters in Fig. S1 (left). To ensure that the wing can be reliably cut on the laser engraver, the airplane geometry is aligned as shown in Fig. S1 (center), with a final fabricated paper airplane shown in Fig. S1 (right). To visually represent the range of geometries that can be formed in our design space a selection of airplane wings are given in Fig. S2.

The robotic system is composed of a number of mechatronic systems which are centrally controlled via a MATLAB control script. The interface and connections between the different components are summarized in Fig. S3. The system relies on accurate trajectory reconstruction. This is achieved with two cameras and a number of vision markers for calibration. This layout is represented in Fig. S4.

S.2. Results

To ensure the optimal number of clusters is selected for the behavioral groups, the strength of clustering is measured when different numbers of clusters are specificed for k-means clustering. In addition to methods outlined in the main text, the elbow method as shown in Fig. S5 is used as heuristics to give confidence to the optimal number of $k = 3$. The system was clustered into three behavioral types. However there are some distinct sub-clusters in the behavioral groups that need to be captured. The need for sub-clustering can also be seen in the weak relationship between distance and geometry parameters expressed as R^2 values in Table S1.

Using the generated cluster-based behavioral model, wing shape optimization experiments are conducted. The flight distances reached and the wing shapes are shown in Fig. S6 for two target flight distances for the model-assisted and unassisted experiments.

Supplementary Videos

Supplementary Video 1 shows the automated fabrication and launch process of the paper airplane using the experimental setup.

Supplementary Video 2 shows the flight trajectory of three different paper airplanes. The system of paper airplanes form behavioral clusters with the representative trajectories being, the nose dive, glide, and recovery glide.
